# Remediation of Soil Mercury by Modified Vermiculite-Montmorillonite and Its Effect on the Growth of *Brassica chinensis* L.

**DOI:** 10.3390/molecules27165340

**Published:** 2022-08-22

**Authors:** Chang Li, Yuchen Li, Hua Cheng, Chunlu Jiang, Liugen Zheng

**Affiliations:** 1 School of Resources and Environmental Engineering, Anhui University, Hefei 230601, China; 2 Anhui Province Engineering Laboratory for Mine Ecological Remediation, Hefei 230601, China

**Keywords:** soil contamination, Hg, modified material, in situ remediation

## Abstract

In this study, the surface of vermiculite-montmorillonite was modified by MnO_2_ loading. The modified vermiculite-montmorillonite was added to remediate the potentially toxic trace element (PTE) Hg present in soil containing coal gangue. Pot experiments were conducted to analyze and compare the pH values, Hg contents and Hg species present in coal gangue-containing soil, with and without the modified materials added, to determine whether the addition of modified materials had an effect on the growth of *Brassica chinensis* L. Results showed that with the addition of 35 g·kg^−1^ modified vermiculite-montmorillonite, the pH of soil increased by a value of 0.79, compared with that in the control group. When 15 g·kg^−1^ was added, the concentration of Hg in soil decreased by 98.2%. The addition of modified materials promoted the transformation of Hg in soil from a bioavailable form to an unavailable form; that is, the content of the residual form increased. The plant height and biomass of *Brassica chinensis* L. also increased, which indicated that the addition of modifiers can increase soil productivity, reduce the effects of PTEs on organisms in soil, and promote plant growth. Therefore, the addition of modified vermiculite-montmorillonite can achieve remediation of coal gangue-containing soil.

## 1. Introduction

Coal gangue is a type of solid waste produced during coal mining, and its production accounts for approximately 10–15% of coal production [1,2]. Coal gangue includes a large number of hazardous substances, and it typically contains the potentially toxic trace element (PTE) Hg [3,4]. Coal mining can cause a series of environmental problems, including subsidence; backfilling with coal gangue is one of the main solutions to this problem. During this process, the presence of PTEs will inevitably cause soil pollution. Mercury is a globally present, persistent and bioaccumulative pollutant. Due to the prevalence of coal-fired power plants, China is the largest source of Hg emissions in the world [5,6]. Hg and its compounds are harmful to human reproduction and the immune and digestive systems [7,8]. Hg can exist in many forms in nature, among which methylmercury is the most toxic. Methylmercury is highly toxic to human nervous, cardiovascular, reproductive, and immune systems [9,10,11,12]. When the concentration of Hg exceeds a certain threshold, it can affect the permeability of plant cell membranes, inhibit the transport of water and nutrients, reduce the efficiency of photosynthesis and respiration, and interfere with enzymatic reactions in plants. Hg disrupts the metabolic processes of plants, resulting in plant dwarfism, leaf withering and, in serious cases, plant death [13]. Hg pollution in the environment mainly originates from the soil matrix, atmospheric deposition, and human activities [14,15,16]. At present, challenges associated with soil Hg pollution exist in many countries, especially in Asian countries such as China, Indonesia, and Malaysia [17]. Therefore, it is particularly important to find and develop effective and sustainable Hg remediation technologies [18,19,20].

According to previous studies, there are two main methods for remediating Hg-contaminated soil: one is to directly reduce the concentration of total Hg in the soil to meet environmental quality limits; and the other is to reduce the risks associated with Hg in soil by changing the existing forms of Hg [21,22]. Remediation techniques for reducing Hg concentrations include heat treatment, chemical leaching, electrochemical treatment, biotechnological treatment (including phytoremediation), vitrification to change the form of Hg, and stabilization and nanotechnology applications [23,24,25]. Although many remediation techniques have been developed, most of them are difficult to apply. In situ passivation technology reduces the risks from pollutants by reducing the mobility of pollutants in soil, and has been widely studied because of its low cost and wide range of applications [26,27]. For Hg, the main remediation mechanism is to fix it to adsorbents by electrostatic forces or via complexation reactions [28], or to convert it into an amalgam by metal precipitation [21]. Clay minerals are a natural, environmentally friendly material with abundant reserves and good adsorption properties [29,30]. Studies have shown that vermiculite and montmorillonite have particularly high passivation effects on PTEs in soil [31,32].

Among various PTE passivation materials, montmorillonite-based mineral materials have been widely studied because of their good adsorption properties, low price, and environmental friendliness [33]. Montmorillonite is an anhydrous aluminosilicate compound with a strong cation exchange capacity and adsorption capacity [34]; it can stabilize and reduce the concentration of PTEs through a variety of reactions, such as coordination, precipitation, and complexation [15]. To improve the ability of montmorillonite to adsorb PTE ions for remediating contaminated soil, many researchers have used a variety of methods to modify montmorillonite [35,36]. MnO_2_ is a manganese oxide that has a large specific surface area and numerous hydroxyl groups on its surface, endowing it with a strong adsorption capacity for pollutants [37,38]. In many studies, MnO_2_ has been loaded directly onto the surface of various materials to increase the specific surface areas and induce stronger adsorption properties in the materials [39,40]. Moreover, manganese dioxide supported on the adsorptive material can generate more hydroxyl groups, or more exchangeable ions, which can be used as adsorbents of PETs and pollutants [41,42,43,44,45].

In this study, MnO_2_ prepared by the reaction of MnSO_4_ and KMnO_4_ was loaded onto vermiculite-montmorillonite, allowing the MnO_2_ to be more firmly bonded to the surface of the material. MnO_2_ was used to modify two types of environmentally friendly clay minerals, and modified vermiculite-montmorillonite was added to soil containing coal gangue. A pot experiment (*Brassica chinensis* L.) was conducted to verify the removal effect of the modified passivator on Hg. The objectives of this study were as follows: (1) to analyze the effects of modified materials on the soil environment; (2) to determine the degree of removal of Hg from coal gangue-containing soil by the modified materials and investigate the resultant effect on plant growth; and (3) to provide a theoretical basis for the remediation of PTEs in soil.

## 2. Materials and Methods

### 2.1. Characteristics of Vermiculite and Montmorillonite

Vermiculite and montmorillonite have large specific surface areas and large ion exchange capacities and show good adsorption of PTE ions [46]. The main component of vermiculite and montmorillonite is SiO_2_, and the two clay minerals have similar structures composed of layered silicates [47,48]. The cation exchange capacities (CECs) of vermiculite and montmorillonite are 0.85 meq·g^−1^ and 0.78 meq·g^−1^, respectively [49]. In addition, there is no Hg present in these minerals.

### 2.2. General Condition of Soil in the Study Area

Soil was collected from the coal mining subsidence area of Linhuan, Huaibei, Anhui Province. Soil samples were collected from five different locations within 100 m^2^. The surface soil samples (0–20 cm) containing coal gangue were collected from the site according to a scheme, and the mixture was uniformly collected in a sample bag to make a representative soil sample. After mixing evenly, the soil was air-dried in a cool and ventilated location, ground and screened, and its physical and chemical properties were determined. The pH value of the soil was determined by a glass electrode method, and the total nitrogen and total phosphorus contents of the soil were determined by the Kjeldahl method and Vanadium molybdate blue colorimetric method, respectively. The results are shown in Table 1.

### 2.3. Modification of Vermiculite-Montmorillonite with MnO_2_

Twenty grams each of vermiculite and montmorillonite was weighed and added to a conical bottle containing 100 mL 2 mol·L^−^^1^ KMnO_4_ solution, and 100 mL 3 mol·L^−1^ MnSO_4_ was added to the conical bottle to form a purple–black powder suspension. The suspension was placed on a shaker at 25 °C and 120 r·min^−1^ for 2 h and then removed and allowed to rest for 24 h. The mixture of vermiculite and montmorillonite was poured into an anhydrous ethanol solution and a certain amount of distilled water was added at a 1:1 ratio and mixed well. The suspension was stirred for 55–65 min, the soaking solution and impurities were poured out and more anhydrous ethanol solution and distilled water were added. This procedure was repeated three times. Then, the mixture of vermiculite and montmorillonite was washed again with distilled water until the pH of the solution was neutral. Finally, the sample was dried in an oven at 70 °C, and the MnO_2_-modified vermiculite-montmorillonite sample was obtained and sealed in a bag for future use.

### 2.4. Pot Experiment

After passing through a 20-mesh sieve, 5.0 kg of farmland soil from a gangue containing area was placed in a clean flowerpot (50 cm × 30 cm × 30 cm), and the modified vermiculite-montmorillonite was added to it. CK was the blank control group, that is, the soil without the modified material. Group A had different amounts of vermiculite-montmorillonite added, group B had different amounts of modified vermiculite-montmorillonite added, and group C had different amounts of MnO_2_-modified vermiculite-montmorillonite added. Group A had 2.5, 5, 15, 25, and 35 g·kg^−1^ vermiculite-montmorillonite added, and group B had 2.5, 5, 15, 25, and 35 g·kg^−1^ modified vermiculite-montmorillonite added. The concentration of MnO_2_ was 3 mol L^−1^ in groups B1, B2, B3, B4, and B5. Group C had modified vermiculite-montmorillonite with MnO_2_ concentrations of 0.2, 0.5, 1.0, 2.0, and 4.5 mol·L^−1^ in groups C1, C2, C3, C4, and C5, respectively, and the amount added was 20 g·kg^−1^.

### 2.5. Sample Collection and Analysis

Plant samples: After the *Brassica chinensis* L. plants had matured for 90 days, 10 healthy plants of the same size were selected from each group to measure the plant height and fresh weight of stems, leaves, and roots. The collected *Brassica chinensis* L. samples were washed with distilled water and dried, deactivated for 40 min in an oven at 105 °C, and dried to a constant weight at 80 °C. The stem and leaf portions were separated from the root portions, ground, and then passed through a 100-mesh nylon sieve. Hg in plant samples was determined by a Hg analyzer (Milestone, DMA-80, Italy). Before each determination, the quartz boat was air-burned so that its absorbance was less than 0.003, and then 0.1 g plant samples were accurately weighed in a quartz boat. The quartz boat was placed in the sample tray of the Hg analyzer and set up according to the program [50].

Soil samples: After the *Brassica chinensis* L. were harvested, the soil samples collected from each group were evenly air-dried, ground and passed through a 100-mesh nylon sieve, and some of the samples were bagged using quadratic sampling (in which all the soil is spread into a circle and divided into four equal parts, then two of these parts are removed; the remaining soil is spread into a circle and divided into four equal parts again, two of the parts are removed, and this operation is repeated until the remaining sample is the required amount for the determination). The PTE species in the soils were analyzed by the Tessier (modified) five-step extraction method [51], in which the metal species are divided into the exchangeable state, carbonate state, Fe-Mn oxidizable state, organic state and residual state, according to the different extraction stages. Using the modified Tessier method, the recovery of Hg as a sum of the fractions relative to THg varied between 86 and 101%, indicating the effectiveness of this approach [52]. The sequential extraction procedure of the Tessier (modified) five-step extraction method is shown in Table 2. The content of Hg was determined directly by a Hg analyzer (Milestone DMA-80, Italy), in which a 0.1 g soil sample was accurately weighed in a quartz boat and the sample was analyzed according to procedure.

## 3. Results and Discussion

### 3.1. Effect of Modified Vermiculite-Montmorillonite on Soil pH

**Figure 1:**
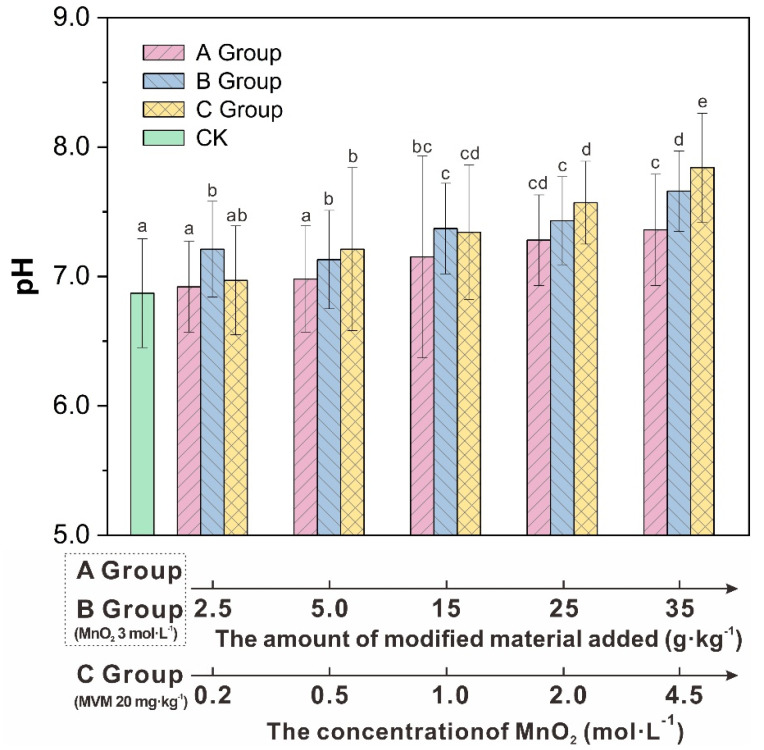
pH values of different groups of soil. (MVM: modified vermiculite-montmorillonite). Different letters above the bar diagram indicate the significant difference of pH in different gruops of soil. The same letters indicate significant differences in means (*p* < 0.05).

The changes in soil pH of the different groups are shown in Figure 1. The results showed that the pH value of the control group was the lowest, and the pH value of the soil in the groups containing different amounts of modified vermiculite-montmorillonite increased to varying degrees with varying amounts of added modifier. The pH value of soil showed an upward trend. In group A, soil pH value for the group containing 35 g·kg^−1^ vermiculite-montmorillonite was the highest, at 7.36, which was 0.49 higher than that of the control group. In group B, the group containing 35 g·kg^−1^ modified vermiculite-montmorillonite had the highest pH value at 7.66, which was 0.79 higher than that of the control group. In group C, the pH value was the highest when the concentration of MnO_2_ was 4.5 mol·L^−1^. The overall pH values of group A, group B and group C were relatively low, indicating that the modified vermiculite-montmorillonite can change the properties of soil to a great extent. The increases in soil pH values caused by the additions of vermiculite-montmorillonite may be due to the large number of soluble base ions, such as Na, K, Ca, and Mg, which are attached to the surface of the vermiculite-montmorillonite modified by MnO_2_. Base ions can exchange with soil hydrogen ions, thus reducing the level of soil hydrogen ions [53]. Hong et al. (2020) [54] added a passivator to soil contaminated with PTEs; the pH of the resulting soil was significantly higher than that of the control group. Sun et al. (2016) [55] added sepiolite to soil, and the pH of soil increased compared with that of the control group. The pH is an important physical and chemical property of soil [56]. In general, the increase in soil pH can promote the formation of PTE carbonate and hydroxide precipitates, thus reducing the biological utilization of PTE [57]. Therefore, the pH value of soil can affect not only the existing forms of PTE in soil, but also the absorption and utilization of nutrients by plants [58,59]. Houben et al. (2013) [60] studied the application of biochar to PTE-contaminated soil and found that, with the increase in biochar application, soil pH increased and the mobility of PTE decreased. Therefore, increasing the pH value of soil is an important way to remediate PTEs pollution.

### 3.2. Remediation of Hg in Soil by Adding Modified Vermiculite-Montmorillonite

The total quantity of PTEs in soil determines the available content of the PTEs, so a change in the total quantity of PTEs will affect the growth of organisms in soil.

**Figure 2:**
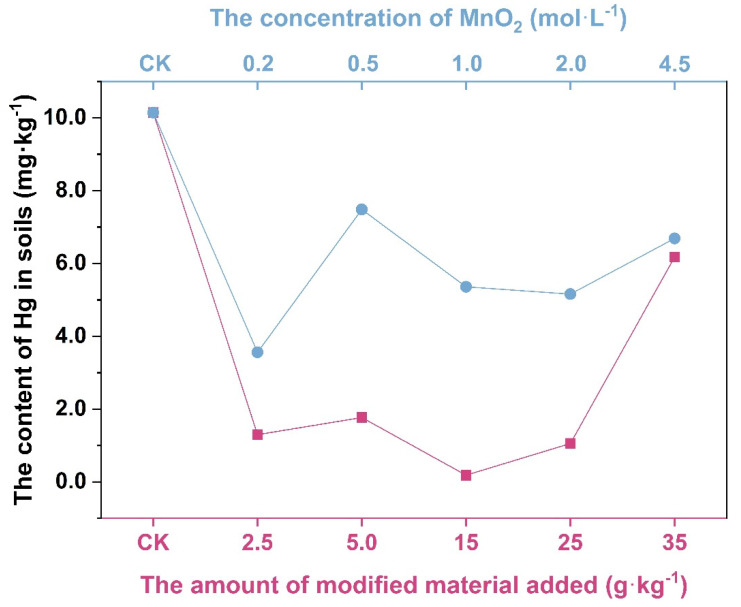
Mercury content of soil modified by different materials, and with different manganese dioxide concentrations.

After the addition of vermiculite-montmorillonite, the concentration of Hg in the soil changed, as shown in Figure 2. Compared with the control group, the content of Hg in the group containing the modifier decreased significantly. When the added amount was the same, the concentration of MnO_2_ was lowest in the 0.2 mol·L^−1^ group. When the concentration of MnO_2_ was the same, the concentration of Hg was lowest in the group containing 15 g·kg^−1^. When the added amount was 2.5 g·kg^−1^, soil Hg concentration decreased by 85.2%, compared with that in the control group. When the added amount was 5 g·kg^−1^, the Hg concentration decreased by 87.59%, and when the added amount was 15 g·kg^−1^, the Hg concentration decreased by 98.2%, which was the greatest change. When the added amount was 25 g·kg^−1^, the Hg concentration in the soil decreased by 89.44%, and when the added amount was 35 g·kg^−1^, the Hg concentration decreased by 39.13%. According to these figures and data, when the amount of modifier added was 15 g·kg^−1^, the remediation effect on soil Hg was the best, while adding greater amounts of the modified amendment led to a decrease in the remediation effect.

**Figure 3:**
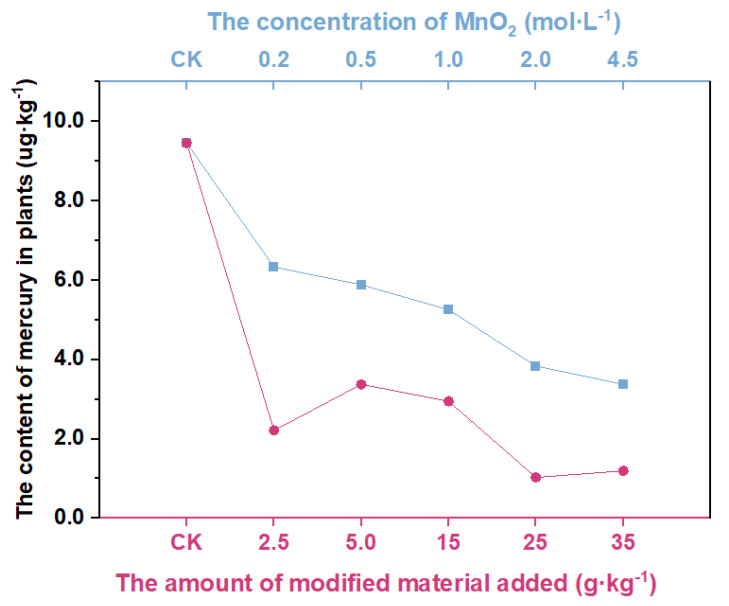
Mercury content of plants modified by different materials, and with different manganese dioxide concentrations.

The effects of different MnO_2_ concentrations of modified vermiculite-montmorillonite, and different amounts of modified vermiculite-montmorillonite on mercury uptake by *Brassica chinensis* L., are shown in Figure 3. Among them, the best remediation effect was when the concentration of MnO_2_ was 25 mol·L^−^^1^, and the content of Hg absorbed by *Brassica chinensis* L. decreased by 89.1%, compared with that in the control group. In group B, with the increase in the amount of modified vermiculite-montmorillonite, the content of mercury absorbed by *Brassica chinensis* L. decreased in turn.

Zhao et al. (2017) [61] used biomass charcoal to repair Hg-contaminated soil; the Hg in the soil was fixed by complexation and precipitation, and the content of Hg in the soil decreased by up to 35%. At the same time, biochar complexed with inorganic Hg to form a relatively stable macromolecular complex, which inhibited the methylation of Hg in the soil and reduced the toxicity of PTEs in the soil. Xia et al. (2021) [62] co-heated corn straw and fly ash to prepare coal ash mixed with biochar composites to remediate soil PTE contamination through surface precipitation, complexation, cation exchange and other interactions to fix soil PTEs, effectively reducing the content of PTEs in the soil. Therefore, vermiculite-montmorillonite shows good Hg removal from soil through complexation and surface adsorption reactions, and the adsorption capacity of modified vermiculite-montmorillonite is even higher. Turull et al. (2019) [63] found that the addition of biochar can reduce the availability of mercury in soil, thereby reducing the absorption of mercury in soil by lettuce. Xing et al. (2019) [64] showed that the addition of rice husk-modified biochar reduced the mobility of mercury in soil, thereby significantly reducing mercury content in various parts of rice, especially in the grains.

### 3.3. Effect of Modified Vermiculite-Montmorillonite on the Hg Species Present in Soil

**Figure 4:**
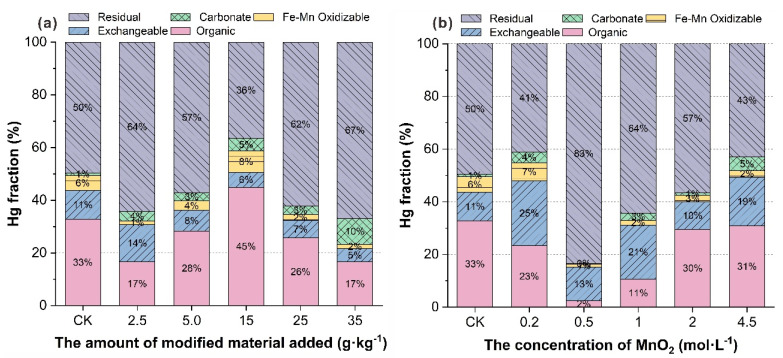
Speciation of mercury in soil modified by different materials, and by different manganese dioxide concentrations.

Adding various amounts of vermiculite-montmorillonite, and vermiculite-montmorillonite produced by different modification conditions, had different effects on the forms of Hg in soil. Figure 4 shows the change in the existing forms of Hg in soil after the addition of quantitatively modified vermiculite-montmorillonite, under different manganese dioxide concentrations. Figure 4b shows that the residual content of Hg in soil modified by 0.5 mol·L^−1^ MnO_2_ was the highest, indicating that the passivation of Hg in soil modified by 0.5 mol·L^−1^ MnO_2_ was the best when other conditions were the same. Additionally, except for the 0.2 and 4.5 mol·L^−1^ groups, the proportion of the residual state increased, compared with the that of control group in each group with vermiculite-montmorillonite added, indicating that the modifier had a certain passivating effect on Hg in soil, and that it can reduce the uptake of Hg by organisms in the soil.

Figure 4a shows the existing forms of Hg in soil after vermiculite-montmorillonite was modified with the same MnO_2_ concentration, and when different amounts of the modifiers were added to the soil. According to Figure 4a, compared with the control group, the proportion of the residual state in each group increased, while the proportion of the exchangeable state and Fe-Mn oxidizable state of Hg decreased in all groups, by 51% and 70%, respectively, except for the 25 g·kg^−1^ group. This may be due to the increase in pH of the soil due to the addition of passivators, such that the relatively active states, such as the exchangeable state in the soil, were fixed by coordination and precipitation reactions into the residual state [65]. It is also possible that the surface of the passivator had pores that allowed the adsorption and transformation of Hg species into the residual state, and fixing them [66]. The results showed that modified vermiculite-montmorillonite can significantly reduce the available Hg content in soil and reduce the risks associated with Hg to the environment and to organisms.

The role of PTEs in the environment is not only related to their total content, but also closely related to their existing forms in the environment [67]. There are also significant differences between the different chemical forms in terms of bioavailability and toxicity [68,69]. Studies have shown that exchangeable and carbonate-bound forms are the most easily absorbed and utilized by plants, and Fe-Mn oxide-bound states represent potentially available states for plants, which can be transformed into a plant-available state under certain conditions [70]. The bioavailability and toxicity of the organic matter binding state is low, so it is not easily absorbed and utilized by plants directly [71], and the residual state is a form that plants cannot absorb and utilize. The interconversion between different forms of Hg in soil is the key process involved in passivation remediation. Therefore, PTE remediation can be achieved by adding a passivator to convert the active forms of Hg in soil into Hg forms that are less active, or are difficult to bio-utilize.

### 3.4. Effect of Modified Vermiculite-Montmorillonite on the Growth of Brassica chinensis L.

The effect of passivators on plant growth, on the one hand, reduces the concentration and toxicity of available PTEs during plant growth; in addition, passivators also provide some elements needed for plant growth, such as silicon and calcium [72].

To more thoroughly explore the effects of modified vermiculite-montmorillonite on soil productivity, the effects of different groups on the growth of *Brassica chinensis* L. (plant height, fresh weight of stems and leaves, dry weight, etc.) were measured in pot experiments after ripening. After maturation, the biomass of *Brassica chinensis* L. in the control group was the lowest. The biomass of the other groups varied based on the type and quantity of materials added.

**Figure 5:**
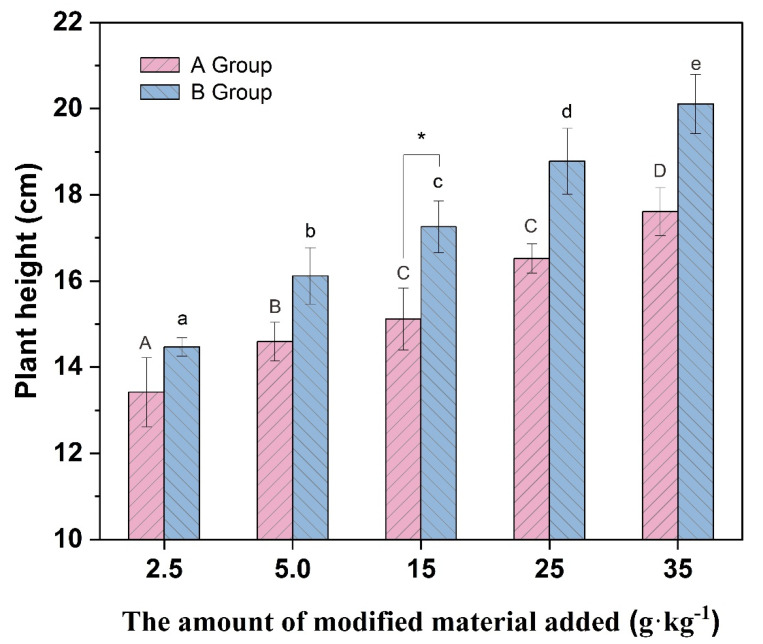
Plant height of different groups of vegetables. (* indicates values significant at *p* < 0.05). Different letters above the bar diagram indicate the significant difference of fresh weight of shoot in different groups of soil.

**Figure 6:**
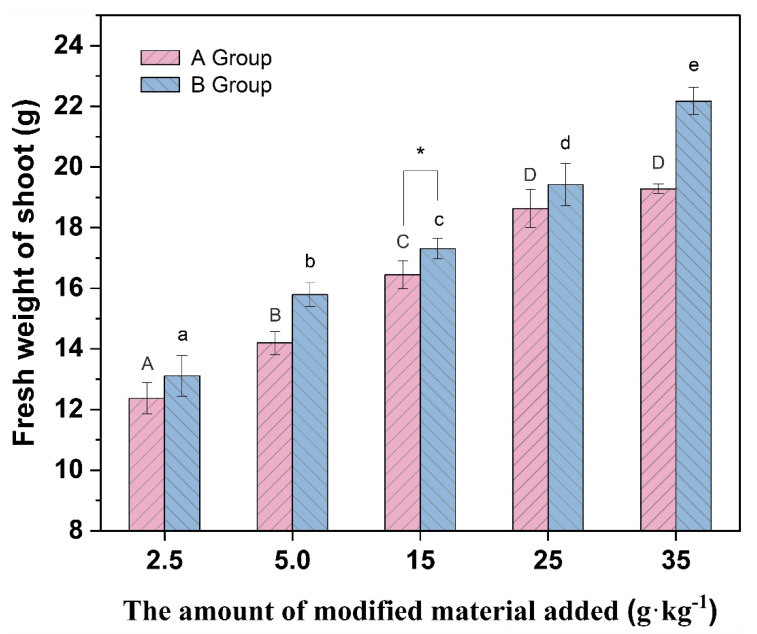
Fresh weight of stems and leaves of different groups of vegetables. (* indicates values significant at *p* < 0.05). Different letters above the bar diagram indicate the significant difference of fresh weight of shoot in different groups of soil.

**Figure 7:**
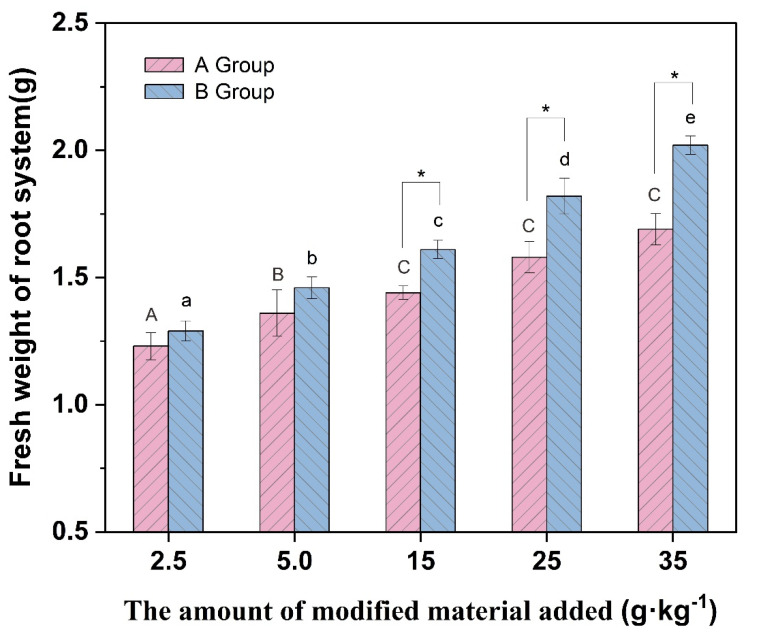
Root fresh weight of different groups of vegetables. (* indicates values significant at *p* < 0.05). Different letters above the bar diagram indicate the significant difference of fresh weight of shoot in different groups of soil.

Figure 5, Figure 6 and Figure 7 show that the plant height and biomass of *Brassica chinensis* L. increased with the increasing addition in each group, potentially because the addition of vermiculite-montmorillonite loosened the soil, absorbed water, and preserved fertilizers, improving the soil by effectively alleviating the toxicity of PTEs to organisms. The results showed that the growth of *Brassica chinensis* L. in group B was higher than that in group A, and when the addition was 15 g·kg^−1^, the plant height and fresh weight of stems and leaves in group B were significantly different from those in group A. The results show that the modified vermiculite-montmorillonite better inhibited the adverse effects of soil PTEs on *Brassica chinensis* L. This may be because after the vermiculite-montmorillonite was modified by MnO_2_, the specific surface area of the material increased, and the loading with MnO_2_ presented more hydroxyl groups that substantially enhanced the PTE adsorption capacity of the material. The absorption of PTEs by plants is affected by different factors, such as the pH, CEC, structure of the soil, available state of PTEs in the soil, and interaction between ions. The addition of passivators can change these factors, thus affecting the absorption of PTEs by plants [73]. In this study, the addition of modified vermiculite-montmorillonite increased the pH of soil, thus reducing the absorption of PTEs by plants. At the same time, the addition of the passivator also reduced the concentration and availability of PTEs to plant roots in the soil, thus promoting plant growth [74].

While the plant height and biomass increased with the increase in vermiculite-montmorillonite addition, there were significant differences in *Brassica chinensis* L. plant height and biomass among the different addition levels. Bashir et al. (2018) [75] studied the effects of different passivators on the absorption of PTEs by hollow vegetables and found that sepiolite could reduce the toxicity of PTEs in soil, promote the growth of hollow vegetables, and fix nutrients. Gu et al. (2020) [76] confirmed that different modifiers had different effects on the biomass of maize, but most of the modifiers significantly increased the aboveground fresh weight and total fresh weight of maize. The above studies show that the modification of vermiculite-montmorillonite can greatly increase soil productivity, reduce the effect of PTEs on organisms, alleviate the effect of metal toxicity on *Brassica chinensis* L., and promote the growth of *Brassica chinensis* L. Xing et al. (2019) [64] showed that adding two different kinds of biochar to contaminated soil can increase the aboveground tissue biomass of rice.

Although the results of this experiment, and other research results, have shown that the addition of modified materials can reduce the toxicity of Hg in soil, the growth of plants is better than that without addition of these materials. However, it cannot be completely ruled out that the addition of modified materials reduces the proportion of Hg in soil, resulting in a decrease in the content of Hg in soil. The added modified material contains macro and trace elements needed by plants, which increases soil fertility and may be one of the reasons for promoting plant growth. In future research, we need to explore which substances in the modified materials play a decisive role in plant growth.

## 4. Conclusions

After vermiculite-montmorillonite was modified by MnO_2_, its specific surface area increased, and its ion exchange capacity was enhanced. Modified vermiculite-montmorillonite is an effective material for removing and passivating PTEs in soil, alleviating toxicity from PTEs, and increasing soil productivity. According to the results of this study, the following conclusions can be drawn: (1) the addition of modified vermiculite-montmorillonite changed the environment of the soil, and the pH of the soil increased; (2) the addition of the passivating materials not only reduced the total content of Hg in soil but also promoted the conversion of Hg from its exchangeable and carbonate-bound forms, which have high bioavailability to its residual form, thereby reducing the availability of PTEs, and the passivators had significant effects on the remediation of PTEs in gangue-containing areas; and (3) the addition of vermiculite-montmorillonite promoted the growth of *Brassica chinensis* L. The plant height and biomass of *Brassica chinensis* L. in group A and group B were significantly greater than those in the control group, and plant growth in group B was significantly better than in group A. These findings show that modified vermiculite-montmorillonite can be effectively applied for remediating PTEs in soil.

## Figures and Tables

**Table 1:** Basic physical and chemical properties of gangue-containing soil.

	pH	Total Nitrogen (g·kg^−1^)	Total Phosphorus (g·kg^−1^)	Organic Matter (g·kg^−1^)
Average Value	6.59	4.81	0.37	40.38

**Table 2:** The sequential extraction procedure of the Tessier (modified) method.

Method and Defined Fractionation	Reagent	Reaction Time and Temperature	Sample/Reagent Ratio (g/mL)
T1:Exchangeable	MgCl_2_ (1 mol·L^−1^ pH = 7)	1 h, r.t. *	1:8
T2:Bound to carbonates	NaAc-Hac (1 mol·L^−1^ pH = 5)	5 h, r.t.	1:8
T3:Bound to iron and manganese oxides	NH_2_OH·HCl (0.04 mol·L^−1^)	6 h, 96 °C	1:20
T4:Bound to organic matter	(1) HNO_3_ + 30% H_2_O_2_ (0.02 mol·L^−1^ pH = 2) (2) 30% H_2_O_2_ (3) NH_4_CH_3_COO (3.2 mol·L^−1^) in 20% HNO_3_	5 h, 85 °C 3 h, 85 °C 0.5 h, r.t.	1:3/1:5 1:3 1:5
T5:Residual	Aqua regia	2 h, 96 °C	1:20

* r.t. = room temperature.

## Data Availability

The datasets used and/or analyzed during the current study are available from the corresponding author on reasonable request.
